# Reliability of quantitative transverse relaxation time mapping with ${\text{T}}_{{2}}$-prepared whole brain pCASL

**DOI:** 10.1038/s41598-020-74680-y

**Published:** 2020-10-27

**Authors:** Martin Schidlowski, Rüdiger Stirnberg, Tony Stöcker, Theodor Rüber

**Affiliations:** 1 Department of Epileptology, University of Bonn Medical Center, Bonn, Germany; 2 German Center for Neurodegenerative Diseases (DZNE), Bonn, Germany; 3 Department for Physics and Astronomy, University of Bonn, Bonn, Germany; 4 Department of Neurology, Epilepsy Center Frankfurt Rhine-Main, Goethe University Frankfurt, Frankfurt/Main, Germany; 5 Center for Personalized Translational Epilepsy Research (CePTER), Goethe University Frankfurt, Frankfurt/Main, Germany

**Keywords:** Biomedical engineering, Magnetic resonance imaging, Brain, Diagnostic markers

## Abstract

Arterial spin labeling (ASL) is increasingly applied for cerebral blood flow mapping, but ${\text{T}}_{{2}}$ relaxation of the ASL signal magnetization is often ignored, although it may be clinically relevant. To investigate the extent, to which quantitative ${\text{T}}_{{2}}$ values in gray matter (GM) obtained by pseudocontinuous ASL (pCASL) perfusion MRI can be reproduced, are reliable and a potential neuroscientific biomarker, a prospective study was performed with ten healthy volunteers (5F,28 ± 3y) at a 3 T scanner. A ${\text{T}}_{{2}}$-prepared pCASL sequence enabled the measurement of quantitative ${\text{T}}_{{2}}$ and perfusion maps. ${\text{T}}_{{2}}$ times were modeled per voxel and analyzed within four GM-regions-of-interest (ROI). The intraclass correlation coefficients (ICCs) of the quantified ASL-${\text{T}}_{{2}}$ varied across brain regions. When averaged across subjects and postlabeling delays (PLDs), the ICCs ranged from reasonable values in parietal regions (ICC = 0.56) to smaller values in frontal regions (ICC = 0.36). Corresponding subject-averaged within-subject coefficients of variation (WSCVs) showed good test–retest measurement precision (${\text{WSCV}}_{{{\text{PLD}}}} \le 0.14$ for all PLDs), but more pronounced inter-subject variance. Reliability and precision of quantified ASL-${\text{T}}_{{2}}$ were region-, PLD- and subject-specific, showing fair to robust results in occipital, parietal and temporal ROIs. The results give rise to consider the method for future cerebral studies, where variable perfusion or altered ${\text{T}}_{{2}}$ times are suspected.

## Introduction

Today, a plethora of quantitative magnetic resonance imaging (MRI) methods is available for scientific research and clinical diagnostics of the brain. These can be categorized into two major groups: structural quantitative MRI, which mainly estimates cerebral tissue parameters, and functional MRI, which allows for measurement of variations in brain activity^1^. Depending on numerous factors, such as MRI pulse sequence, measurement protocol, data processing and analysis, quantitative MRI methods result in (semi-)quantitative parameter maps given in physical units, which are intercomparable^1–3^. Compared to typical, non-quantitative MR imaging, these values allow the user to infer the cerebral status and brain characteristics, potentially longitudinally. The usefulness and suitability of such parameters for diagnostic tests depend on the accuracy, the precision and the reliability of the applied method^4^.

Perfusion imaging is a functional quantitative method widely established in clinical routine^5^. Commonly used quantities in brain perfusion MRI are (relative) cerebral blood flow ((r)CBF) and volume ((r)CBV) as well as the transfer constant ${\text{k}}_{{{\text{trans}}}}$. The former two are usually assessed by dynamic susceptibility contrast (DSC) MRI and latter by dynamic contrast enhanced (DCE) MRI. In both cases exogenous, often gadolinium-(Gd-)based contrast agents are applied^2, 3^.

Arterial spin labeling (ASL) is an alternative perfusion technique, which uses blood water molecules as an endogenous tracer instead of injection of exogenous contrast agents. It provides arterial transit time (ATT) in addition to cerebral blood flow (CBF) and has been applied in practice for a comparatively short time^6^. The main advantages of ASL are its non-invasiveness and a fairly simple processing for quantification, making it suitable for daily scanning routine^7, 8^.

The endogenous tracer is created by labeling, which is the inversion of arterial blood water magnetization. This can be accomplished by three alternative main techniques:^9, 10^ Continuous ASL inverts spins within a defined slice by applying a steady labeling pulse, whereas pulsed ASL inverts spins within a whole slab once per imaging repetition time. Pseudocontinuous ASL is another approach utilizing a train of rapidly repeating short labeling pulses, thereby imitating the continuous approach without the need for special hardware for constant radiofrequency transmission.

The tracer creation is followed by a delay prior to data acquisition. At the time of measurement, the previously inverted spins have not reached the state of equilibrium which results in a reduced signal. Based on this, the ASL label is primarily determined by the longitudinal $\text{T}_{1}$ decay, whereas the perfusion-weighted ASL signal also depends on perfusion, blood volume and arrival times^11, 12^. The related quantities CBF and ATT are derived voxel-wise and measured frequently both in scientific and clinical practice. However, the transverse relaxation time (${\text{T}}_{{2}}$) of the ASL signal magnetization has yet received little attention. Previous studies have shown, that labeled arterial protons flow into cerebral structures, where an exchange of water molecules takes place between intravascular (IV) and extravascular (EV) space. When the labeled protons cross the semipermeable blood–brain barrier, the observed ${\text{T}}_{{2}}$ values change from blood-${\text{T}}_{{2}}$ to tissue-${\text{T}}_{{2}}$^13–15^. Furthermore, several studies show distinct disease-related ${\text{T}}_{{2}}$ changes in conventional ${\text{T}}_{{2}}$-weighted, non-perfusion-weighted images, which enable classification of brain pathologies, such as tumor progression^16, 17^. A recent study demonstrates the use of ${\text{T}}_{{2}}$-weighted ASL in an animal model of blood–brain barrier dysfunction^18^. Also, the impact of physiologic alterations on the cerebral perfusion and ${\text{O}}_{{2}}$-metabolism seems to correlate with a change of both CBF and ${\text{T}}_{{2}}$^19^. Therefore, ${\text{T}}_{{2}}$ of the ASL signal might be a parameter of interest, but the potential and suitability is unknown. In this work, the reliability and precision of current state-of-the-art ASL-${\text{T}}_{{2}}$ measurements are investigated, laying the ground for future research.

## Results

### Subjects and pCASL parameters

The study included ten participants of balanced gender distribution (5M, 5F) with an average age of 28 ± 3 years. The interscan latency between measurements was 24 ± 15 days and subject specific scanner SAR rates for the pCASL sequence were 72 ± 17 %. The labeling slab was positioned individually and adjusted via the distance to the lower edge of the imaging volume with an average of 31 ± 8 mm.

### Perfusion

**Figure 1:**
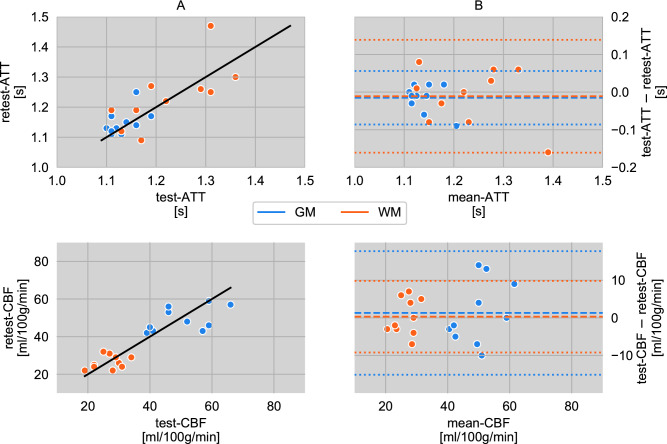
(**A**) Correlation of cerebral blood flow (CBF) and arterial transit time (ATT) from repeated measurements of ten subjects in gray and white matter. The solid line is the identity function and represents optimal correlation. (**B**) Agreement (Bland–Altman plot) between test and retest CBF and ATT from values shown in (**A**). The mean differences between both scans in gray and white matter are represented by the dashed line and dotted lines display the corresponding limits of agreement ($1.96 \times$ standard deviations of mean differences).

The gray matter (GM) and white matter (WM) CBF and ATT values of the retest measurements are plotted versus the corresponding values of the test measurements in Fig. 1A. Corresponding Bland–Altman plots are provided in Fig. 1B. The mean CBFs are 27 ml/100 g/min for WM and 50 ml/100 g/min for GM with test–retest intraclass correlation coefficients (ICCs) of 0.37 (WM) and 0.49 (GM), respectively. Both cerebral components are equal in their within-subject coefficient of variation (WSCV) of 0.12. Likewise, the WM/GM-results for ATT estimates are: average ATTs of 1.23 s/1.14 s, ICCs of 0.71/0.47 and WSCVs of 0.04/0.02. The group statistics are summarized in Table 1 together with the respective standard deviation. The limits of agreement for CBF and ATT measurements are displayed in the Bland–Altman plot in Fig. 1B.

**Table 1:** Perfusion parameter results. Mean arterial transit time (ATT [s]) and cerebral blood flow (CBF [ml/100 g/min]) and statistical test–retest results (ICC, WSCV, SDD) in white (WM) and gray matter (GM).

parameter	ROI	test	retest	ICC	WSCV	SDD
ATT	GM	1.13 ± 0.03	1.15 ± 0.04	0.47	0.02	0.07
ATT	WM	1.23 ± 0.09	1.24 ± 0.11	0.71	0.04	0.14
CBF	GM	50.5 ± 9.44	49.2 ± 6.46	0.49	0.12	16
CBF	WM	26.7 ± 4.67	26.4 ± 3.63	0.37	0.12	9

**Figure 2:**
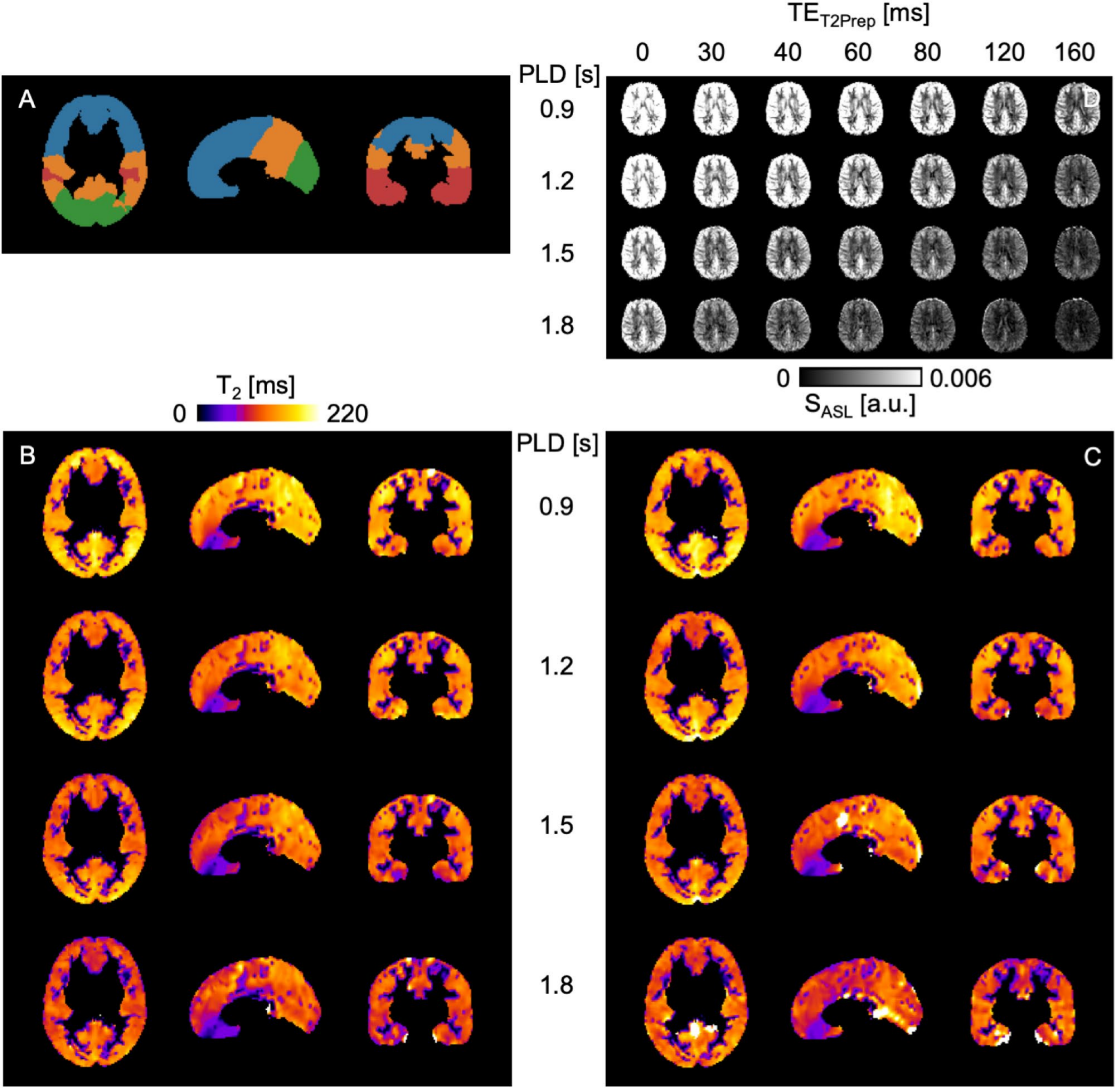
(**A**) Regions of interest (ROIs) of the MNI reference map: frontal (blue), occipital (green), parietal (orange), temporal (red) and corresponding masked gray matter ${\text{T}}_{{2}}$ maps in MNI space for all postlabeling delays PLD = 0.9/1.2/1.5/1.8 s for (**B**) test and (**C**) retest scans of one single subject. (**D**) Mean perfusion-weighted, non-smoothed whole brain ASL signal maps in native space for all PLDs and ${\text{T}}_{{2}}$ preparation times ${\text{TE}}_{{{\text{T2Prep}}}}$ = 0/30/40/60/80/120/160 ms.

### ${\text{T}}_{{2}}$quantification

**Figure 3:**
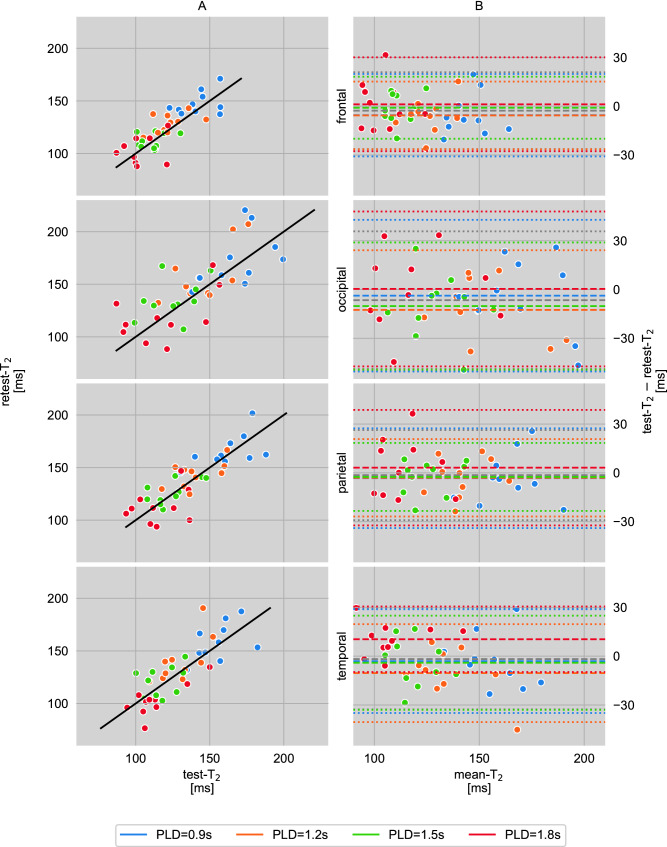
(**A**) Correlation of median ${\text{T}}_{{2}}$ values from repeated measurements of ten subjects in four gray matter regions of interest (ROIs) at four postlabeling delays PLD = 0.9/1.2/1.5/1.8 s. The solid line is the identity function and represents optimal correlation. (**B**) Agreement (Bland–Altman plot) between test and retest ${\text{T}}_{{2}}$ values which are shown in (**A**). The mean differences between both scans at each PLD are represented by the dashed lines and dotted lines display the corresponding limits of agreement ($1.96 \times$ standard deviations of mean differences).

Two representative ${\text{T}}_{{2}}$ maps, which have been obtained with Eq. (2), are shown in Fig. 2B for test measurements, and in Fig. 2C for retest measurements of one single subject for all PLDs. Median ${\text{T}}_{{2}}$ values in four GM regions of interest (ROIs), according to Fig. 2A, are shown in Fig. 3A for all ten participants and all PLDs of both measurements separately. The corresponding Bland–Altman plots are presented in Fig. 3B.

**Figure 4:**
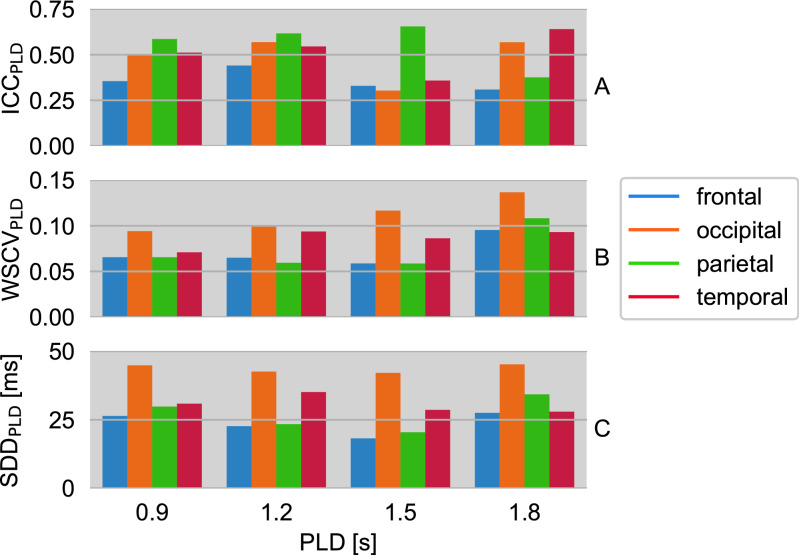
(**A**) Intraclass correlation coefficient ${\text{ICC}}_{{{\text{PLD}}}}$, (**B**) within-subject coefficient of variation ${\text{WSCV}}_{{{\text{PLD}}}}$ and (**C**) smallest detectable difference ${\text{SDD}}_{{{\text{PLD}}}}$ of median ${\text{T}}_{{2}}$ values from repeated measurements of ten subjects. Results are shown for four postlabeling delays PLD = 0.9/1.2/1.5/1.8 s and four gray matter regions of interest (ROIs).

**Figure 5:**
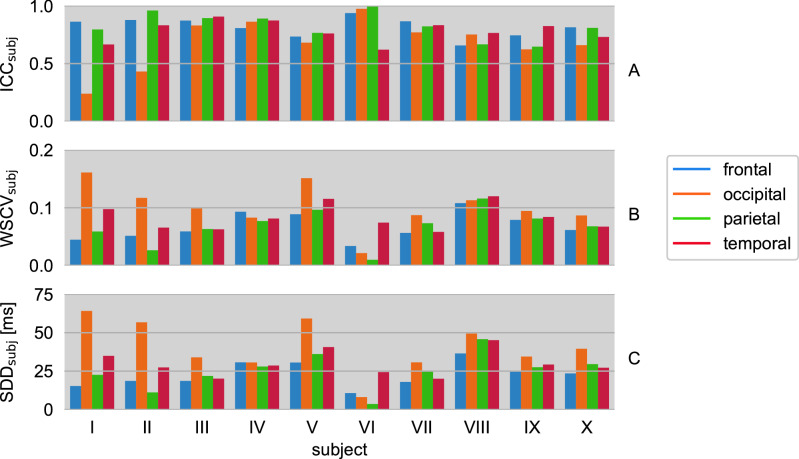
(**A**) Intraclass correlation coefficient ${\text{ICC}}_{{{\text{subj}}}}$, (**B**) within-subject coefficient of variation ${\text{WSCV}}_{{{\text{subj}}}}$ and (**C**) smallest detectable difference ${\text{SDD}}_{{{\text{subj}}}}$ of median ${\text{T}}_{{2}}$ values from repeated measurements at four postlabeling delays PLD = 0.9/1.2/1.5/1.8 s. Results of all ten subjects are shown for four gray matter regions of interest (ROIs).

Subject-averaged ${\text{ICC}}_{{{\text{PLD}}}}$ values are displayed in Fig. 4A. Averaging over all PLDs yields the following mean ICCs per ROI: frontal $\overline{{{\text{ICC}}}}_{{{\text{PLD}}}}$ = 0.36, occipital $\overline{{{\text{ICC}}}}_{{{\text{PLD}}}}$ = 0.49, parietal $\overline{{{\text{ICC}}}}_{{{\text{PLD}}}}$ = 0.56 and temporal $\overline{{{\text{ICC}}}}_{{{\text{PLD}}}}$ = 0.52. The corresponding PLD-averaged ${\text{ICC}}_{{{\text{subj}}}}$ results are presented in Fig. 5A.

According to Fig. 4B, subject-averaged ${\text{WSCV}}_{{{\text{PLD}}}} \le 0.14$ for all PLDs and ROIs. Corresponding PLD-averaged ${\text{WSCV}}_{{{\text{subj}}}} \le 0.16$ are shown in Fig. 5B for all subjects.

The smallest detectable differences (SDDs) averaged across all subjects ${\text{SDD}}_{{{\text{PLD}}}}$ are presented in Fig. 4C. These are 24 ms in the frontal, 44 ms in the occipital, 27 ms in the parietal, and 31 ms in the temporal ROI. The SDDs are also shown as the limits of agreement in the Bland–Altman plots (Fig. 3B). Corresponding PLD-averaged ${\text{SDD}}_{{{\text{subj}}}}$ are shown in Fig. 5C.

## Discussion

In this study, ten subjects were scanned with a ${\text{T}}_{{2}}$-prepared pCASL sequence and the data obtained were used for a quantification of ASL-${\text{T}}_{{2}}$ values. Since little is known about this quantity, we have investigated whether it is precise and reproducible. The results revealed a robustness that makes further research and potentially clinical application of ASL-based ${\text{T}}_{{2}}$ measurements promising. At the same time, it becomes clear that not only the ${\text{T}}_{{2}}$ values, but also their reliabilities dependent on the subject, the observed cerebral structure (ROI) and the selected postlabeling delay (PLD).

The measured perfusion parameters, CBF and ATT, are in accordance with common literature values^11^. For CBF there is a fair test–retest reliability in GM and a poor one in WM, while ATT estimation is relatively precise in both GM and WM. Besides the commonly observed biological variability of CBF^20, 21^ an additional possible reason for the comparably low ICCs in this study might be that only the non-${\text{T}}_{{2}}$-weighted images were used for perfusion parameter calculation. Those images correspond to only 14 % of the data collected in total and were thus acquired in a relatively short time of ~ 6.5 min^22, 23^. Furthermore, the non-${\text{T}}_{{2}}$-weighted data were sporadically acquired over an exceptionally long time (~ 45 min), such that motion artifacts and perfusion changes may have an increased influence. The ATTs show test–retest reliabilities, which are also fair in GM and according to the classifications can be rated as good in WM. Interestingly, the ATT ICC values are higher in WM than in GM. As the study focused on determining ${\text{T}}_{{2}}$ of the perfusion-weighted ASL signal, no ${\text{T}}_{{2}}$-weighted ${\text{M}}_{{0}}$ calibration images have been acquired, in line with common ASL practice. For a CBF modeling including ${\text{T}}_{{2}}$-weighted data, however, those calibration images are required, which remains to be examined in the future. In order to still analyze the impact of the ${\text{T}}_{{2}}$-weighted images on perfusion modeling, additional CBF and ATT calculations were performed by first generating mean perfusion-weighted images separately per ${\text{T}}_{{2}}$ weighting. Subsequently, each image obtained was scaled so that its mean was equal to the corresponding non-${\text{T}}_{{2}}$-weighted image mean. Finally, an averaged image with improved SNR was generated from all adjusted images and the non-${\text{T}}_{{2}}$-weighted image. While CBF and ATT as well as their related WSCVs and SDDs showed only minor changes, the ICCs remarkably improved as summarized in Table 2.

**Table 2:** Perfusion parameter results of the extended data analysis including all ${\text{T}}_{{2}}$-weighted scans after previous normalization to the mean signal of the conventional non-${\text{T}}_{{2}}$-weighted image per ${\text{TE}}_{{{\text{T2Prep}}}}$. Mean arterial transit time (ATT [s]) and cerebral blood flow (CBF [ml/100 g/min]) and statistical test–retest results (ICC, WSCV, SDD) in white (WM) and gray matter (GM).

parameter	ROI	test	retest	ICC	WSCV	SDD
ATT	GM	1.10 ± 0.04	1.10 ± 0.06	0.55	0.02	0.08
ATT	WM	1.20 ± 0.12	1.21 ± 0.13	0.74	0.05	0.16
CBF	GM	48.9 ± 8.81	46.2 ± 7.25	0.63	0.10	13
CBF	WM	26.5 ± 4.57	25.8 ± 4.33	0.50	0.11	8

Averaged median ${\text{T}}_{{2}}$ values for perfusion-weighted ASL signal and ASL control signal are shown in Supplementary Information Fig. S1. In the test–retest study, medium reliabilities of the quantified ASL-${\text{T}}_{{2}}$ data were found in healthy young adults. ${\text{ICC}}_{{{\text{PLD}}}}$ show the highest (fair) test–retest reliability in the parietal ROI followed by the temporal and the occipital ROI. The frontal ROI is to be considered as least reliable ROI (poor). A differentiation between the four PLDs leads to a good reliability of the parietal ROI at the shorter PLDs, whereas the ${\text{ICC}}_{{{\text{PLD}}}}$ of the temporal ROI tends to increase with PLD, apart from PLD = 1.5 s. In this context, the occipital and frontal ROIs indicate no distinct PLD dependence. One possible explanation for the low frontal reliability may be that this largest ROI includes areas directly above the nasal cavities. Strong ${\text{B}}_{{0}}$ inhomogeneities in that area, which is notoriously difficult to shim with conventional shim coils, causes inter-voxel dephasing and poor signal-to-noise ratio. According to the example data shown in Fig. 2, this can locally result in reduced measured ${\text{T}}_{{2}}$ across all PLDs. An additional analysis of the frontal ROI with a caudal-cranial subdivision, however, does not confirm that the caudal segment yields particularly poor PLD-specific reliability metrics across subjects (see Supplementary Information Figs. S2 and S3). On the contrary, the cranial segment seems to drive the poor overall frontal reliability, in particular at long PLDs. An analysis of the single subject parameters reveals a homogeneous distribution of ${\text{ICC}}_{{{\text{subj}}}}$ within the individual ROIs, except for the occipital ROI in two participants.

Generally, there is a moderate precision, ${\text{WSCV}}_{{{\text{PLD}}}} \le 0.14$, for all PLDs and ROIs with the highest precision in the frontal and parietal ROIs and the lowest precision in the occipital ROI. A loss of precision in ${\text{T}}_{{2}}$ measurement seems to occur at the longest PLD. ${\text{WSCV}}_{{{\text{subj}}}}$ analysis reveals strong differences between subjects and frequently elevated values in the occipital ROI.

With regard to PLD-dependence, the smallest detectable differences ${\text{SDD}}_{{{\text{PLD}}}}$ share the same characteristics as the WSCVs. Importantly, the bulk ${\text{T}}_{{2}}$ estimates decrease with increasing PLD in all ROIs. One-way analysis of variance (ANOVA) with repeated measures indicates significant differences in ${\text{T}}_{{2}}$ of the PLDs among each other (P < 0.001) except for measurements at PLD = 1.5 s compared to those at PLD = 1.8 s (P = 0.183). This strongly supports that measuring ${\text{T}}_{{2}}$-prepared pCASL at different labeling and postlabeling delays may be a useful strategy, and that the corresponding bulk ${\text{T}}_{{2}}$ may be a useful biomarker. For instance, there is already evidence for brain tumor classification utilizing conventional ${\text{T}}_{{2}}$ relaxometrie^16, 17^. Furthermore, a differentiation of glioblastomas multiforme, metastases and primary CNS lymphomas with Gadolinium-based DSC perfusion-weighted imaging based on perfusion differences in the tumor core respectively the peritumoral zone has been demonstrated^24^. The transverse relaxation time of blood water protons depends to some extent on the surrounding tissue properties and also on the blood oxygenation level. Likewise, the tissue characteristics and vascularization change in tumors, thereby impacting the blood oxygenation status and tissue perfusion^25,26^. It may be presumed that the ${\text{T}}_{{2}}$-weighted ASL signal will be also altered and therefore ${\text{T}}_{{2}}$, combined with the perfusion parameters CBF and ATT, might provide a non-invasive basis for a comprehensive model for disease characterization.

Since the presented technique for determining ${\text{T}}_{{2}}$ is still under investigation, this study has some limitations and potential sources of error in ${\text{T}}_{{2}}$ quantification. Physiological fluctuations can be minimized but not completely suppressed by background saturation and may disturb the ASL data^11, 27^. In addition, the patient positioning and labeling alignment may affect the measurements twofold:^28^ First, an unfavorable placement of the frequency-sensitive and vessel-flow-dependent labeling slab leads to a lower ASL signal and thus to potential CBF underestimations. Second, different labeling alignments between scans lead to a decrease in test–retest reproducibility and precision. In this study, minimizing the latter error source was attempted by using screenshots to recall labeling positions by a single operator. An advanced automatic positioning method would be highly desirable. However, current automatic positioning methods are often not suited for optimal labeling alignment in ASL. Nevertheless, assuming common arterial blood flow velocities (~ 20 cm/s^11^), the wide variance in applied imaging-labeling gaps and the related time it takes the labeled blood to arrive in brain areas, should not have any measurable impact on ${\text{T}}_{{2}}$. Generally, a low SNR can lead to inaccuracies and reduced precision at the parameter fitting stage, but also during preprocessing, in particular motion correction and normalization. One option to achieve better SNR per image or more SNR-equivalent images in the same scan time in parallel imaging, is to employ CAIPIRINHA instead of conventional GRAPPA sampling^27, 29^. Unfortunately, the scanner software used in this study did not allow for online reconstruction of CAIPIRINHA data. However, given that only a parallel imaging acceleration factor of two was used, the expected gain in SNR (reduced geometry factor^30^) when compared to GRAPPA would have been small. The use of parallel imaging and a twofold segmented readout reduced the echo train length. Still remaining blurring can be considered to be of equal extent throughout the ${\text{T}}_{{2}}$ weightings as the preparation does not affect the 3D GRASE readout.

For future research, it is important to consider that SNR-limited ASL data require repeated measurements to boost statistical power for CBF and ATT quantification, and also a reasonable number of ${\text{T}}_{{2}}$ weightings to fit the data to the ${\text{T}}_{{2}}$ relaxation model (Eq. 2). Even longer scan times would improve ${\text{T}}_{{2}}$ fit quality. To nevertheless increase the clinical relevance and to obtain a practicable measurement protocol, a suitable attempt might be to reduce the number of different PLDs or possibly to focus on a single PLD. As each PLD comprises all ${\text{T}}_{{2}}$ weightings but is unrelated to each other, an omission of a certain PLD, for instance, would reduce Fig. 2B–C by one row. Accordingly, acquiring multi-${\text{T}}_{{2}}$-weighted ASL data at only one PLD with the protocol of this study would correspond to only approximately 13 min of measurement time, depending on the PLD. Also, a shortening can be achieved by reducing the number of ${\text{T}}_{{2}}$ weightings. A choice of 4, for example, would save about 43 % of time. However, this would also degrade ${\text{T}}_{{2}}$ fit quality as it is reflected by poorer ${\text{ICC}}_{{{\text{PLD}}}}$, ${\text{WSCV}}_{{{\text{PLD}}}}$ and ${\text{SDD}}_{{{\text{PLD}}}}$ especially at longer PLDs (see Supplementary Information Fig. S4).

Despite the limitations mentioned, our results already show a suitability to use this method in practice at the current stage of development. The reliability and precision results do not indicate an obvious recommendation for a specific PLD to be measured. However, it can be assumed that a proper choice depends to some extent on the study objectives and measurement conditions. For example: in order to investigate an accumulation of the labeled blood, as in tumors, a sufficiently long PLD should be chosen to allow a transition into the pathological tissue.

Altogether, assessed precision and reproducibilities of the ASL signal ${\text{T}}_{{2}}$ times allow to conclude, that ASL-${\text{T}}_{{2}}$ is an interesting and promising metric to investigate cerebral abnormalities and a potential marker for disease-related ${\text{T}}_{{2}}$ changes, presumably due to blood–brain barrier dysfunction^14, 18^. For instance, the ${\text{T}}_{{2}}$-prepared pCASL method may prove useful to classify brain tumors, where altered perfusion or ${\text{T}}_{{2}}$ times can be expected as previously shown in in conventional ${\text{T}}_{{2}}$-weighted images^16, 31^. This approach allows to avoid the use of contrast agents, which not only ensures absolute non-invasiveness^7, 11^, but also bypasses methodological limitations of current contrast agent-based methods. Its clinical relevance remains to be investigated in future research.

## Material and methods

### Study design and experiments

**Figure 6:**
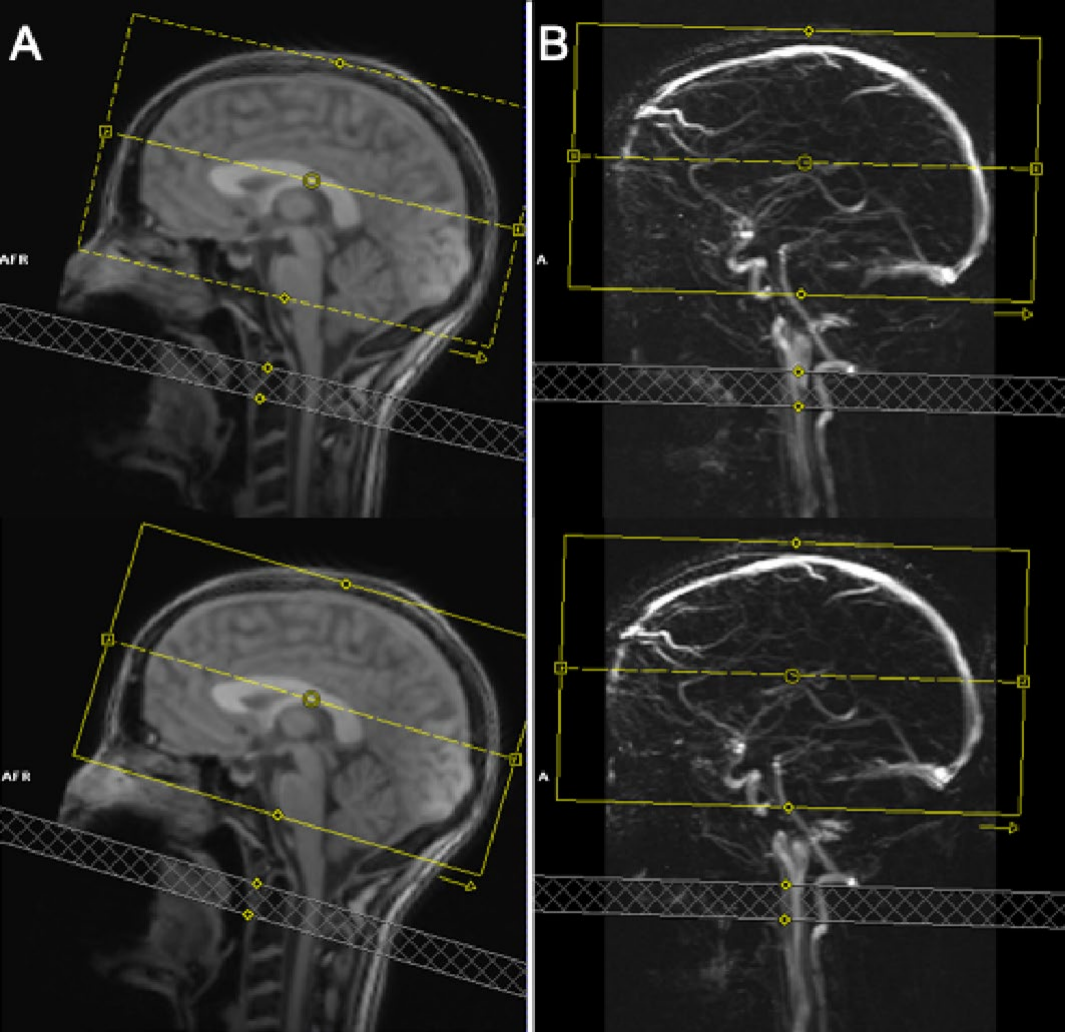
(**A**) Structural localizer and (**B**) time-of-flight (TOF) vessel scout for placement and alignment of the labeling volume (gray hatched) in test (top) and retest (bottom) scan to invert blood in basilar, right and left internal carotid arteries. The same gap to the image volume (yellow) was used in both measurements performed by the same operator.

Ten young healthy volunteers (5 female, 28 ± 3 years) without known neurologic and psychiatric disorders and free from drug or alcohol abuse were scanned on a Magnetom Trio 3 T scanner (Siemens Healthineers, Erlangen, Germany) using a 32-channel head coil. This study was approved by the institutional review board of the medical faculty of the University of Bonn. All experiments were performed in accordance with the guidelines and regulations of this ethics board. Written informed consent was obtained from all subjects. In order not to affect the CBF, the participants were asked to abstain from nicotine one hour before measurements and caffeine three hours before measurements^32, 33^. Test and retest experiments, with an interscan latency of at least two weeks, were performed at about the same time of day to reduce the impact of circadian rhythms. Following the “auto-align” anatomical localizer scout, a time-of-flight vessel scout was acquired. The latter was used to position the ASL labeling plane slightly inferior to the lower end of the cerebellum by adjusting the gap between the lower edge of the ASL imaging volume and the parallel labeling plane. This ensures an inversion of blood water magnetization in the basilar and the right and left internal carotid arteries^28^. For every subject, a screenshot of the ASL planning has been taken during the first scan. The same gap has been applied in the second scan as to minimize label placement influences and to correct for changes in head position between both sessions (Fig. 6). Adjustments of the automatic MRI planning, and in particular the alignment of the ASL plane, was carried out by a single operator in all measurements to minimize variance of the operator bias.

The acquisition protocol and ASL parameters are summarized in Table 3. In brief, the voxel size of the twofold segmented 3D GRASE readout^27^ was 3 × 3 × 4 mm^3^ and an unbalanced pCASL labeling with a labeling duration of 1.8 s, four postlabeling delays PLD = 0.9/1.2/1.5/1.8 s and seven ${\text{T}}_{{2}}$ preparation lengths ${\text{TE}}_{{{\text{T2Prep}}}}$ = 0/30/40/60/80/120/160 ms was applied. Each control-tag (CT) pair and segment (SEG) was measured six times for each PLD and each ${\text{TE}}_{{{\text{T2Prep}}}}$, resulting in a scan time of 45:20 min. Proton density-weighted calibration images ${\text{M}}_{{0}}$ were acquired without background suppression and a longer TR of 6 s. The otherwise same readout, differed in an alternating orientation of the phase encoding (PE) polarity between each of four measurements (1:15 min in total). This allowed for geometric distortion estimation and correction in post processing.

**Table 3:** Acquisition protocol and ASL parameters. Calibration images, ${\text{M}}_{{0}}$, were acquired with alternating PE polarity and distortion corrected using FSL’s function topup. ${\text{M}}_{{0}}$ and ASL data were acquired with a twofold segmented (2 × SEG) 3D GRASE readout. Additionally, a structural ${\text{T}}_{{1}}$-weighted image (resolution (0.8 mm)$^{3}$) was measured. The scan times were 45:20 min for ASL data (6 MEAS/PLD/${\text{TE}}_{{{\text{T2Prep}}}}$/SEG/CT pair), 1:15 min for corresponding calibration scans (2 MEAS/PE-direction) and 6:32 min for the ${\text{T}}_{{1}}$ scan resulting in a total scan time of 53:07 min. Abbreviations: ASL, Arterial Spin Labeling; BW, Bandwidth; ETL, Echo Train Length; FOV, Field Of View; PE, Phase Encoding; PLD, Postlabeling Delay; SEG, Segment; TE, Echo Time; TR, Repetition Time.

3D GRASE		ASL	
FOV	210 × 210 × 120 mm^3^	LD	1.8 s
acquisition matrix	70 × 70 × 30	PLD	0.9/1.2/1.5/1.8 s
resolution	3 × 3 × 4 mm^3^	${\text{TE}}_{{{\text{T2Prep}}}}$	0/30/40/60/80/120/160 ms
${\text{TE}}_{{{\text{GRASE}}}}$	22.9 ms	TR	4.02 s
PE	GRAPPA 2x		
3D	2 × SEG		
BW	2551 Hz/Px	**M**_**0**_ **calibration**	
ETL	355 ms	TR	6 s

Additionally, a structural ${\text{T}}_{{1}}$-weighted image with an isotropic resolution of 0.8 mm was acquired (6:32 min) in the first (test) session. In sum, the ASL protocol with the calibration and structural scans corresponded to a total measurement time of 53:07 min.

### pCASL sequence

**Figure 7:**
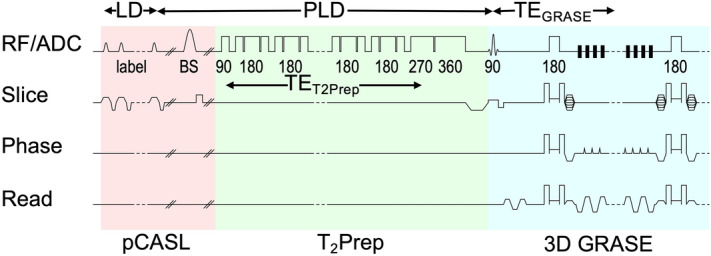
Diagram of ${\text{T}}_{{2}}$ prepared pCASL sequence. A WET saturation of the imaging region is performed before pCASL labeling. Three background suppression (BS) pulses are timed to suppress static tissue signal. Only a single BS pulse is shown for clarity. The pulses may intersperse with the labeling pulses and thus interrupt the pCASL labeling but not the ${\text{T}}_{{2}}$ preparation which is played out immediately before the 3D GRASE readout. In case of an interruption the label condition is changed between tag and control. In order to induce different ${\text{T}}_{{2}}$ weightings, ${\text{TE}}_{{{\text{T2Prep}}}}$, the preparation module is composed of non-selective pulses arranged in three different Malcolm Levitt (MLEV) cycling schemes with 4 (${\text{TE}}_{{{\text{T2Prep}}}}$ = 30/40 ms), 8 (${\text{TE}}_{{{\text{T2Prep}}}}$ = 60/80 ms) and 16 (${\text{TE}}_{{{\text{T2Prep}}}}$ = 120/160 ms) composed refocusing pulses as well as a composed 270°-360° tip-up pulse to compensate for off-resonance effects and ${\text{B}}_{{1}}$ imperfections. Abbreviations: ADC, Analog-To-Digital Conversion; BS, Background Suppression; LD, Labeling Duration; pCASL, Pseudocontinuous Arterial Spin Labeling; PLD, Postlabeling Delay; RF, Radio Frequency (Transmission); TE, Echo Time.

An in-house developed ${\text{T}}_{{2}}$-prepared pCASL sequence^27, 34^ provides multi ${\text{T}}_{{2}}$ weighting and multi postlabeling delay data. A schematic sequence diagram is given in Fig. 7. The preparation module consists of non-selective composite pulses arranged in different Malcolm Levitt (MLEV)^35^ cycling schemes with varying numbers of refocusing pulses to create ${\text{T}}_{{2}}$ weightings with varying echo times ${\text{TE}}_{{{\text{T2Prep}}}}$. By using this method, off-resonance effects are reduced, ${\text{B}}_{{1}}$ imperfections compensated and transverse relaxation time estimates improved^15, 36^. Images were reconstructed online using the generic GRAPPA^37^ implementation on the scanner.

### Data preprocessing

Preprocessing included the application of toolboxes from *FMRIB’s Software Library*^38^ (FSL, v6.0.3, https://fsl.fmrib.ox.ac.uk/fsl/fslwiki). The ASL data were first corrected for motion using *mcflirt*^39^ followed by distortion correction utilizing *topup*^40^ on the basis of the calibration scans with alternating PE polarity. Then, mean perfusion images were computed for every PLD and ${\text{TE}}_{{{\text{T2Prep}}}}$ by a pairwise subtraction of CT pairs:

1 $$S_{{{\text{ASL}}}} \left( {{\text{PLD}},{\text{TE}}_{{{\text{T2Prep}}}} } \right) = \left( {M_{{{\text{control}}}} \left( {{\text{PLD}},{\text{TE}}_{{{\text{T2Prep}}}} } \right) - M_{{{\text{tag}}}} \left( {{\text{PLD}},{\text{TE}}_{{{\text{T2Prep}}}} } \right)} \right)/M_{{0}} .$$

A brain mask was generated based on the corrected calibration scans using FSL’s brain extraction tool *bet*^41^. In order to obtain the normalization warp field from structural to MNI space, the ${\text{T}}_{{1}}$-weighted structural scan was processed with *fsl_anat*^42^.

### Data analysis

CBF and ATT were calculated using the FSL tool *oxford_asl*^43^. Both preprocessed ASL data and *fsl_anat* results were taken as the input. Thus, firstly a GM mask was generated, and secondly the ASL data were coregistered to structural space as well as normalized to MNI space.

For ${\text{T}}_{{2}}$ mapping, the preprocessed perfusion-weighted mean difference images were first multiplied by the binary GM mask and then smoothed with an isotropic Gaussian filter kernel ($\sigma_{{{\text{Gauss}}}}$ = 2 mm, FWHM = 4.7 mm). Subsequently, ${\text{T}}_{{2}}$ values and the extrapolated baseline signal at TE = 0 ms ($S_{{0}} \left( {{\text{PLD}}} \right)$) were calculated for each postlabeling delay and for each voxel by applying a monoexponential non-linear least squares (NLLS) fit along ${\text{TE}}_{{{\text{T2Prep}}}}$ using the python function *curve_fit* from the *Scipy.Optimize* library (v1.4.1, https://www.scipy.org):

2 $$S_{{{\text{ASL}}}} \left( {{\text{PLD}},{\text{TE}}_{{{\text{T2Prep}}}} } \right) = S_{{0}} \left( {{\text{PLD}}} \right) \cdot e^{{ - {\text{TE}}_{{{\text{T2Prep}}}} /T_{{2}} }} .$$

The derived ${\text{T}}_{{2}}$ values correspond to the bulk ${\text{T}}_{{2}}$ of the ASL signal magnetization within a voxel at a particular postlabeling delay. Finally, the ${\text{T}}_{{2}}$ maps were spatially normalized using the previously calculated transformations.

### Statistical analysis

The determined CBF, ATT and ${\text{T}}_{{2}}$ values were statistically analyzed by performing computations with the package *agRee*^44^ (v0.5–2) from the *R project*^45^ *(v3.6.2, *https://www.r-project.org*).*

The longitudinal test–retest reliability was assessed by using the intraclass correlation coefficient (ICC) for one-way random-effects ANOVA model as proposed by Shrout and Fleiss^46–49^. Qualitatively, a higher ICC, which can range from 0 to 1, indicates more reliable results. For quantitative assessments, the following value ranges were considered:^50^

- poor: < 0.4
- fair: 0.4–0.59
- good: 0.6–0.74
- excellent: > 0.75

Furthermore, Bland–Altman plots were created, which can be taken for visual review of the reliabilities^4^.

The measurement precision, which reflects the variability and the measurement error, was evaluated according to the within-subject coefficient of variation (WSCV)^47, 51, 52^. It quantifies the errors relative to the size of the measurements and is calculated by a division of the within-subject standard deviation by the global mean^53^. Small values correspond to high precision.

A third parameter utilized for statistical evaluation of the experiments is the smallest detectable difference (SDD). It is also based on a one-way random-effects ANOVA and is defined as the 100(1 − α/2) % quantile of the distribution of test–retest differences^54^, thus representing a large proportion of typical test–retest differences. Here, this proportion equals 95 % according to the selected confidence level of the interval, α = 0.1. The SDDs are shown as “limits of agreement” in the Bland–Altman plots. Small SDDs are preferable.

For a ROI-based statistical analysis of test–retest ${\text{T}}_{{2}}$ quantifications, previously normalized ${\text{T}}_{{2}}$ maps in the MNI space were taken and evaluated in four GM ROIs obtained from the MNI structural atlas (2 mm, zero thresholded)^55, 56^ provided with FSL, namely frontal, occipital, parietal and temporal ROI (Fig. 2A). For this purpose, the median ${\text{T}}_{{2}}$ values within the individual ROIs were determined and assigned to test and retest groups for ten subjects with each of four PLDs. Based on these median scan and rescan ${\text{T}}_{{2}}$ values, statistical parameters were calculated in two different ways: (a) across subjects at every PLD and (b) across PLDs for every single subject. According to method (a) parameters ${\text{ICC}}_{{{\text{PLD}}}}$, ${\text{WSCV}}_{{{\text{PLD}}}}$ and ${\text{SDD}}_{{{\text{PLD}}}}$ were derived and according to method (b) the parameters ${\text{ICC}}_{{{\text{subj}}}}$, ${\text{WSCV}}_{{{\text{subj}}}}$ and ${\text{SDD}}_{{{\text{subj}}}}$ were obtained analogously. The statistical evaluation of ATTs and CBFs compared GM and WM ROIs only.

## Supplementary information

Supplementary Information 1

## Data Availability

Study data are not publicly available in order to respect data protection and privacy of the participants. Further information can be requested from the corresponding author.
