# Supplementary material for: Reliability of quantitative transverse relaxation time mapping with T2-prepared whole brain pCASL
Source: Sci Rep. 2020 Oct 27;10:18299. doi: 10.1038/s41598-020-74680-y (PMC7592060; doi:10.1038/s41598-020-74680-y)
Supplement: Supplementary file 1 — Supplementary Information 1 [file 41598_2020_74680_MOESM1_ESM.pdf]

Reliability of Quantitative Transverse Relaxation Time Mapping with T_2-Prepared Whole Brain pCASL

Martin Schidlowski[1,2], Rüdiger Stirnberg[2], Tony Stöcker[2,3,*], Theodor Rüber[1,4,5,*]

[1]Department of Epileptology, University of Bonn Medical Center, Bonn, Germany

[2]German Center for Neurodegenerative Diseases (DZNE), Bonn, Germany

[3]Department for Physics and Astronomy, University of Bonn, Bonn, Germany

[4]Epilepsy Center Frankfurt Rhine-Main, Department of Neurology, Goethe University Frankfurt, Frankfurt/Main, Germany

[5]Center for Personalized Translational Epilepsy Research (CePTER), Goethe University Frankfurt, Frankfurt/Main, Germany

[*]These authors contributed equally to this work.

Supplementary Figure S1

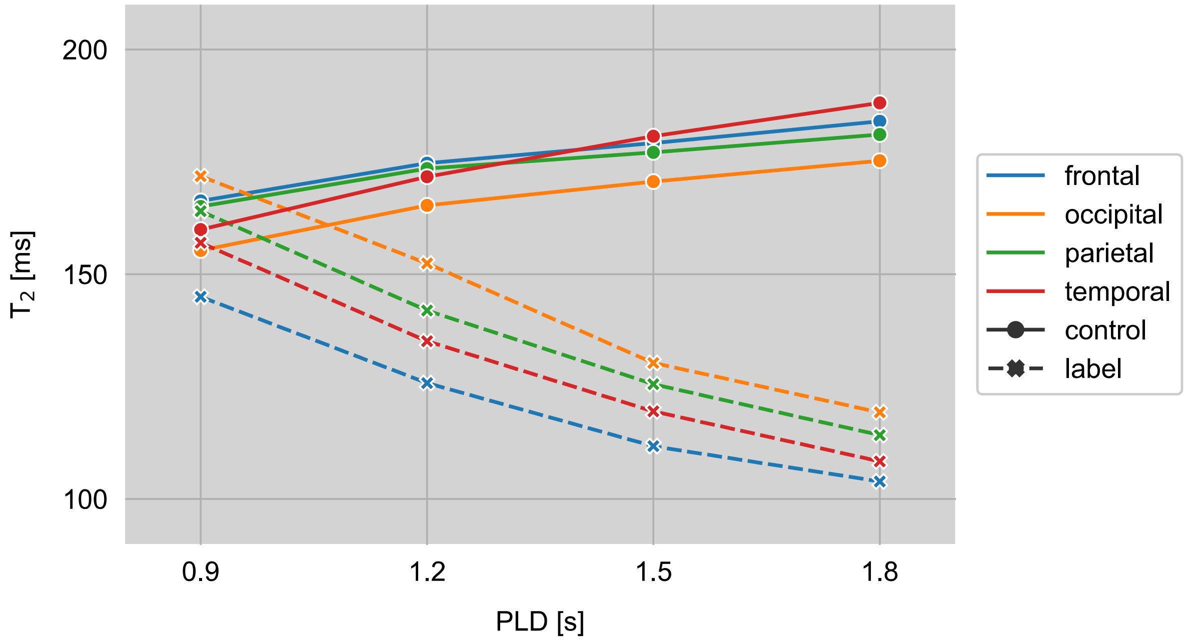

Averaged median T_2 values of perfusion-weighted ASL signal (solid) and ASL control signal (dashed) from repeated measurements of ten subjects at four postlabeling delays PLD=0.9/1.2/1.5/1.8 s in four gray matter regions of interest (ROIs).

Supplementary Figure S2

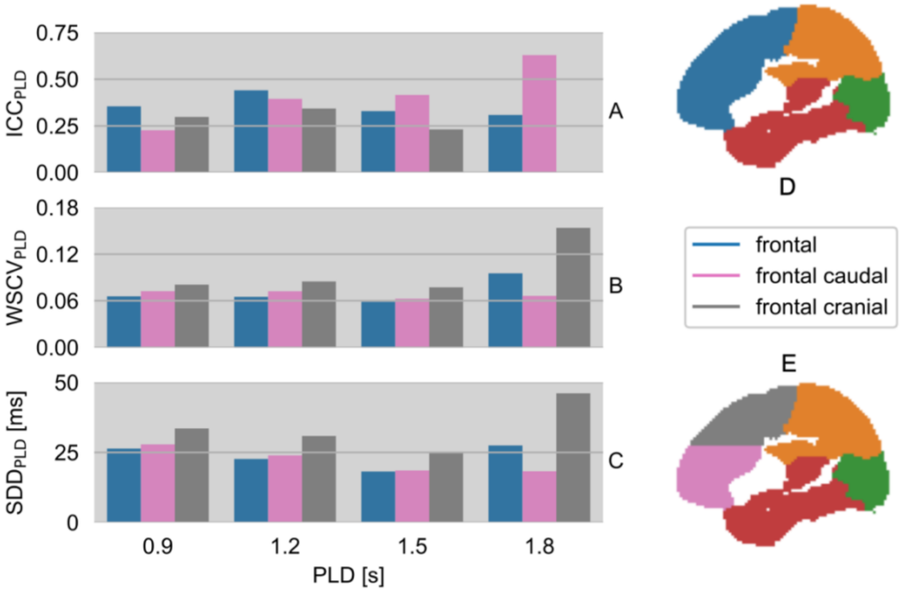

(A) Intraclass correlation coefficient ICC_{PLD}, (B) within-subject coefficient of variation $WSCV_{PLD}$ and (C) smallest detectable difference SDD_{PLD} of median T_2 values from repeated measurements of ten subjects. Results are shown for four postlabeling delays PLD=0.9/1.2/1.5/1.8 s in frontal gray matter regions of interest (ROIs). The frontal ROI (blue in D) corresponds to the region used for the previous statistical analysis. This region was subdivided into a caudal (pink) and a cranial (gray) segment according to (E). The frontal caudal segment, which suffers from susceptibility-induced T_2 reduction, does not yield poor measurement reliability than the large frontal ROI. Interestingly, however, the frontal cranial segment yields a considerably poorer measurement reliability, in particular at longer PLDs. For comparison, the same analysis without the two most unreliable subjects (V, VIII) provides a frontal-cranial ICC_{PLD} of 0.37 at PLD=1.8 s.

Supplementary Figure S3

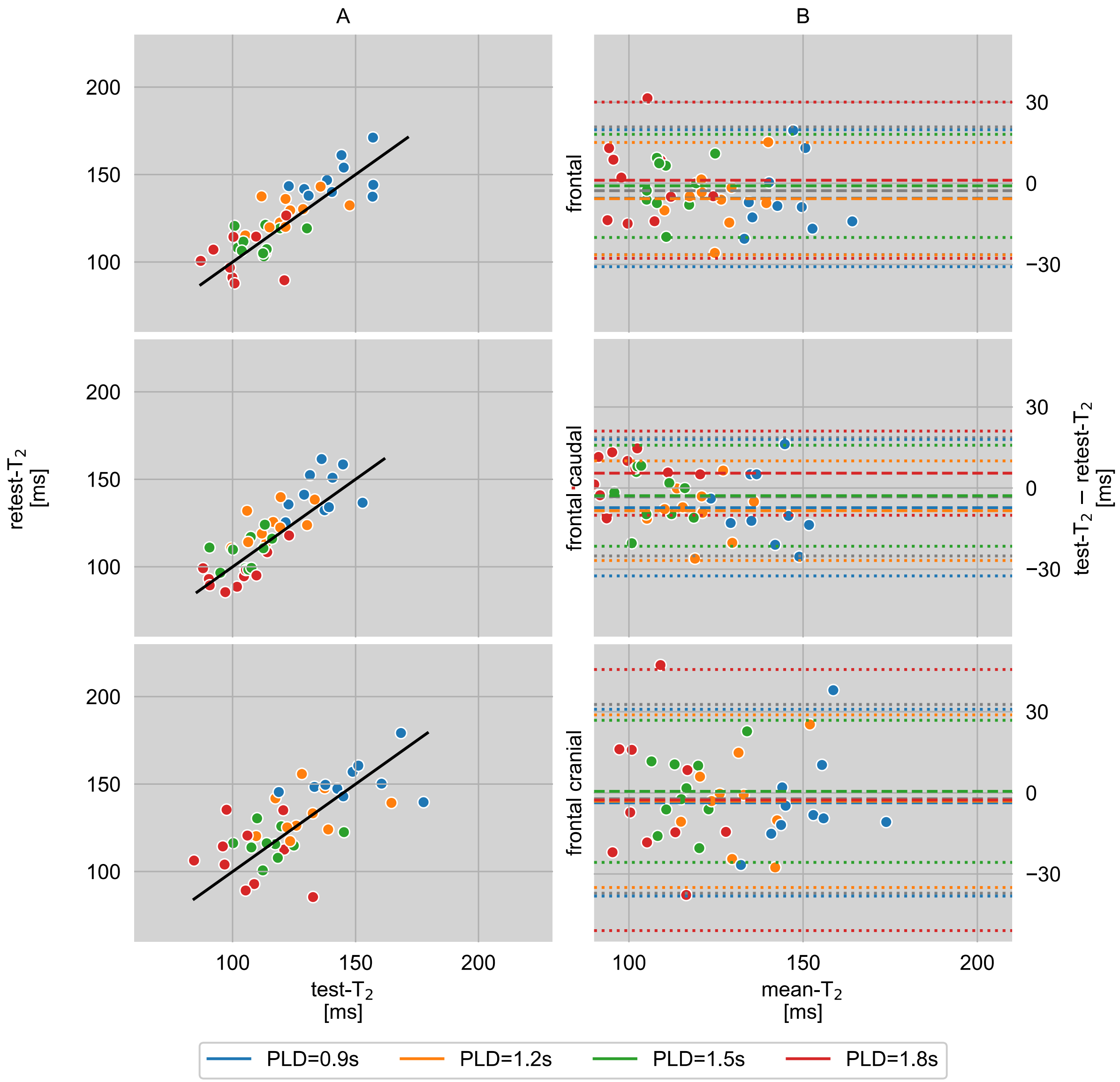

(A) Correlation of median T_2 values from repeated measurements of ten subjects in frontal gray matter regions of interest (ROIs) at four postlabeling delays PLD=0.9/1.2/1.5/1.8 s. The subdivision of the frontal ROI into a cranial and a caudal segment is shown in Supplementary Figure S2E. The solid line is the identity function and represents optimal correlation. (B) Agreement (Bland-Altman plot) between test and retest T_2 values which are shown in (A). The mean differences between both scans at each PLD are represented by the dashed lines and dotted lines display the corresponding limits of agreement ($1.96 \times$ standard deviations of mean differences).

Supplementary Figure S4

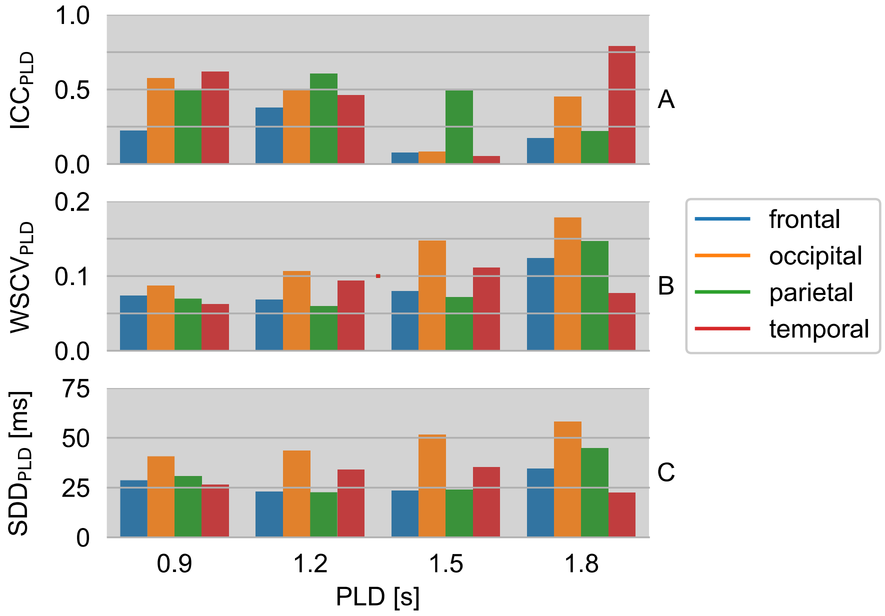

(A) Intraclass correlation coefficient ICC_{PLD}, (B) within-subject coefficient of variation $WSCV_{PLD}$ and (C) smallest detectable difference SDD_{PLD} of median T_2 values from repeated measurements of ten subjects based on a reduced T_2 preparation time subset TE_{T2Prep}=0/40/80/160 ms. Results are shown for four postlabeling delays PLD=0.9/1.2/1.5/1.8 s and four gray matter regions of interest (ROIs).
